# Endoscopic Versus Surgical Management for Infected Necrotizing Pancreatitis and Walled-Off Necrosis: A Systematic Review of Randomized Controlled Trials

**DOI:** 10.3390/medicina61122149

**Published:** 2025-12-02

**Authors:** Manuela Mastronardi, Giada Moghnie, Sara Crociato, Chiara Menghini, Alessio Biagio Filippo Giordano, Paola Germani, Margherita Sandano, Nicolò de Manzini, Alan Biloslavo

**Affiliations:** 1 Department of Medicine, Surgery and Health Sciences, University of Trieste, 34129 Trieste, Italy; 2 General Surgery Unit, Cattinara University Hospital, Azienda Sanitaria Universitaria Giuliano Isontina, 34148 Trieste, Italy; 3 Emergency Surgery Unit, Careggi University Hospital, Largo Brambilla 3, 50134 Florence, Italy; 4 Department of Surgery, San Carlo Borromeo Hospital, Azienda Socio-Sanitaria Territoriale Santi Paolo e Carlo, 20142 Milan, Italy

**Keywords:** acute pancreatitis, necrotizing pancreatitis, endoscopic necrosectomy, minimally invasive surgery, VARD

## Abstract

*Background and Objectives*: Infected necrotizing pancreatitis and walled-off necrosis are associated with substantial morbidity and mortality. The evolution from open necrosectomy to minimally invasive and endoscopic strategies has improved outcomes, yet complex cases may require multimodal approaches. *Materials and Methods*: A systematic literature search was performed across PubMed, Web of Science, and Google Scholar from inception to 15 July 2024, following PRISMA 2020 guidelines. Only RCTs directly comparing endoscopic and surgical necrosectomy were included. We analyzed RCTs enrolling adults with infected necrotizing pancreatitis or symptomatic/infected walled-off necrosis, irrespective of etiology, comparing endoscopic step-up strategies with surgical or minimally invasive step-up approaches. Outcomes assessed included mortality, complications, hospital stay, long-term pancreatic function, and quality of life. *Results*: Six RCTs comprising 1045 patients were identified. Endoscopic necrosectomy demonstrated comparable mortality to surgical or minimally invasive step-up approaches (8–18% vs. 6–15%) but significantly reduced rates of pancreatic fistula (8% vs. 34%, *p* < 0.01), new-onset organ failure, and, in several studies, shortened hospital stay. Median timing of intervention ranged from 4 to 6 weeks after pancreatitis onset, although some trials reported earlier or delayed drainage, highlighting variability in clinical practice. Long-term endocrine and exocrine pancreatic function, as well as quality of life, were largely similar between techniques, although early recovery and physical functioning scores favored endoscopy in selected studies. *Conclusions*: Endoscopic necrosectomy offers a safer peri-procedural profile compared with surgical approaches, but complex or anatomically unfavorable necrosis may still require surgical intervention. Individualized multimodal management, informed by evolving evidence, represents the cornerstone of modern care for patients with infected necrotizing pancreatitis and walled-off necrosis.

## 1. Introduction

Acute necrotizing pancreatitis is a severe and potentially fatal condition that develops in up to 20% of patients with acute pancreatitis [1]. Infected necrosis occurs in approximately one-third of cases and is associated with high morbidity and mortality [2]. Historically, open necrosectomy was considered the standard of care, but it carried substantial risks, including multiple organ failure, pancreatic and enterocutaneous fistulas, long-term pancreatic insufficiency, and impaired quality of life [3,4].

The management of necrotizing pancreatitis has evolved markedly over the past two decades. The introduction of the step-up approach—beginning with percutaneous catheter drainage followed, if necessary, by minimally invasive surgical debridement—demonstrated improved outcomes compared with upfront open necrosectomy [4,5,6]. Parallel to this, endoscopic ultrasound (EUS)-guided drainage and endoscopic necrosectomy have emerged as less invasive alternatives, with growing evidence suggesting comparable or superior short-term outcomes, particularly regarding fistula formation, recovery time, and quality of life [7,8].

Despite these advances, clinical practice is often complex. Patients may require escalation from one modality to another, and in selected cases, a combined endoscopic and surgical approach is needed to achieve definitive control of infection and resolution of necrosis [1,9]. This reflects the need to integrate evidence-based strategies into individualized care. This systematic review aims to synthesize randomized evidence comparing endoscopic and surgical necrosectomy in infected or walled-off necrosis, focusing on mortality, complications, and functional outcomes.

## 2. Materials and Methods

This review was conducted in accordance with the PRISMA 2020 guidelines [10]. Only RCTs that met the following criteria were included in the study:

- Population: Adults with infected necrotizing pancreatitis or symptomatic/infected walled-off necrosis
- Intervention: endoscopic step-up approach (drainage ± necrosectomy)
- Comparison: surgical/minimally invasive step-up approach (percutaneous drainage ± VARD/laparoscopic necrosectomy)
- Outcomes: Major complications, mortality, length of stay (LOS), time to resolution of necrosis, long-term complications (endocrine or exocrine insufficiency, recurrent pancreatitis), and quality of life (QoL).
- Design: Randomized controlled trials only.

The included randomized trials enrolled adults with infected necrotizing pancreatitis or available in this field include patients with infected necrotizing pancreatitis and walled-off necrosis of mixed etiologies, and our review was reframed accordingly to reflect the actual populations studied.

A systematic search of PubMed, Web of Science and Google Scholar was performed from inception to 15 July 2024. For each database, we used a deliberately broad search string to ensure maximal sensitivity and avoid missing eligible randomized controlled trials. The exact Boolean string applied was:

“necrotizing pancreatitis OR walled-off pancreatic necrosis AND necrosectomy OR VARD OR Video-Assisted Retroperitoneal Debridement OR endoscopic drainage OR percutaneous drainage AND randomized controlled trial AND english”.

The search was executed using the default Boolean precedence rules of each database (i.e., automatic interpretation of AND/OR operators), as recommended when conducting highly sensitive exploratory searches. No filters other than language (English) were applied. This broad strategy was intentional to capture all potentially relevant studies before applying strict eligibility criteria during screening. The complete search strategy was developed in collaboration with a medical librarian to ensure methodological rigor.

Only RCTs published in English were considered. Reference lists of included trials and relevant reviews were also screened to identify additional eligible studies. Two reviewers independently screened titles, abstracts, and full texts for eligibility. Discrepancies were resolved by consensus. Data extracted included: author, year, study design, recruitment period, population, interventions, comparisons, outcomes, complications, mortality, LOS, time to resolution of necrosis, long-term pancreatic complications, and quality of life measures.

Two independent reviewers assessed the risk of bias of each of the six included randomized controlled trials (RCTs). We used the revised Cochrane Risk-of-Bias Tool for Randomized Trials (RoB 2) framework [11], which evaluates bias across five core domains: (1) bias arising from the randomization process, (2) bias due to deviations from intended interventions, (3) bias due to missing outcome data, (4) bias in measurement of the outcome, and (5) bias in selection of the reported result. Within each domain, signaling questions were answered to inform the judgment of ‘low risk’, ‘some concerns’, or ‘high risk’ of bias. Where disagreements arose, they were resolved by discussion or by consulting a third reviewer. For each trial we recorded both domain-specific judgments and an overall risk-of-bias judgment (the most adverse domain rating determines the overall rating).

Visual “traffic-light” plots were generated to summarize judgments.

Statistical Analysis

No new statistical analyses were performed. Data from the included randomized controlled trials were extracted and summarized in tabular form. The main outcomes assessed were mortality, length of hospital stay, post-procedural complications, long-term pancreatic insufficiency, and quality of life. Where available, relative risks (RRs), confidence intervals (CIs), and *p*-values were reported as provided in the original publications. Owing to heterogeneity in study design, populations, and outcome definitions, a quantitative meta-analysis was not performed.

## 3. Results

**Figure 1:**
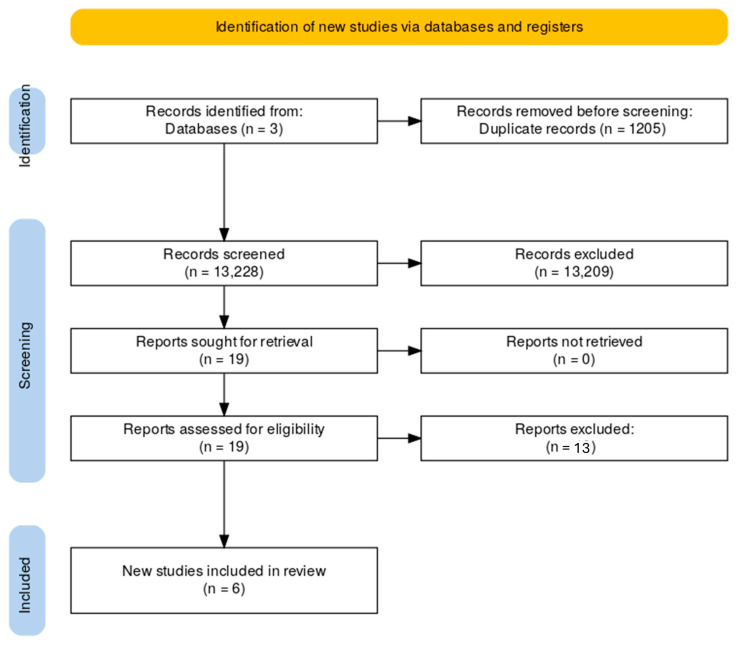
PRISMA 2020 flow diagram showing the process of study identification, screening, eligibility assessment, and inclusion of randomized controlled trials.

A systematic literature search was conducted in accordance with the PRISMA 2020 guidelines [10]. The initial search identified 597 records from PubMed, 12,316 from Web of Science, and 1520 from Google Scholar (Figure 1). After removal of duplicates and screening for relevance, only six RCTs met the inclusion criteria and were included in the final analysis.

**Figure 2:**
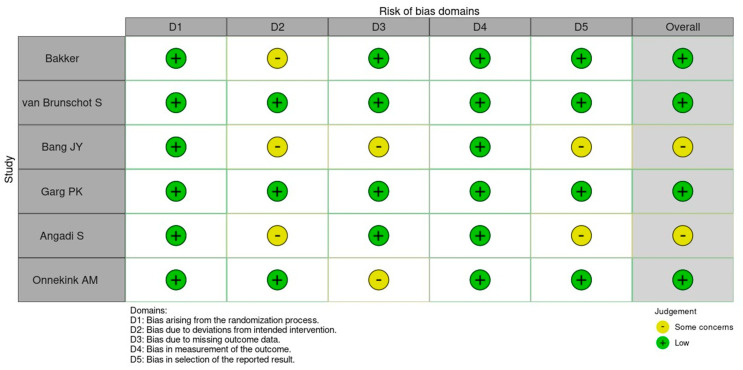
Risk-of-bias assessment of included randomized controlled trials using the Cochrane RoB 2 tool. Domains: D1 = randomization process; D2 = deviations from intended interventions; D3 = missing outcome data; D4 = measurement of outcomes; D5 = selection of reported results. Green = low risk; yellow = some concerns [8,12,13,14,15,16].

The risk of bias of the six included randomized controlled trials was evaluated using the Cochrane RoB 2 tool [11]. Overall, four studies were judged to be at low risk of bias across all domains (Bakker et al. [12], van Brunschot et al. [13], Garg et al. [14], and Onnekink et al. [8]), while two studies presented some concerns [15,16]. No study was considered at high risk of bias in any domain (Figure 2).

These studies (Table 1) evaluated the comparative effectiveness of endoscopic necrosetomy (EN) versus surgical or minimally invasive surgical necrosectomy (SN/MISN) in patients with infected necrotizing pancreatitis or symptomatic walled-off necrosis (WON). The included trials were conducted between 2008 and 2022, with sample sizes ranging from 34 to 418 patients. In several trials, the total study population refers to the number of patients screened or enrolled in the institutional cohort, whereas only a subset of these patients underwent randomization to endoscopic or surgical step-up interventions. For clarity, we report both the overall study population and the exact number of randomized patients in Table 1.

Across studies, interventions compared EN, step-up endoscopic strategies, and minimally invasive or open surgical drainage, with primary outcomes focused on major complications, organ failure, or mortality.

Across all included randomized controlled trials, interventions were typically delayed allowing maturation of the necrotic wall. In line with international guidelines, drainage or necrosectomy was performed at least four weeks after disease onset in most studies. Van Brunschot et al. [13] reported a median interval of 39–41 days between symptom onset and the first intervention, while Bang et al. [15] found similar medians of 35 and 38 days for the endoscopic and surgical groups, respectively. Onnekink et al. [8] reported a median treatment duration of 17 days for endoscopic and 41 days for surgical interventions after randomization, corresponding approximately to 2–6 weeks and 6–20 weeks from diagnosis. Angadi et al. [16] included only patients with walled-off necrosis present for more than four weeks, ensuring a matured capsule before intervention. Bakker et al. [12] and Garg et al. [14] showed that most drainage procedures occurred between 4 and 6 weeks and, in later-stage cases, up to 14–22 weeks after pancreatitis onset.

Overall, these findings confirm a consistent delay in intervention timing, with most procedures performed around the fifth to sixth week after the onset of acute pancreatitis, avoiding early drainage (<4 weeks) unless clinically indicated.

Post-procedural morbidity was generally lower in the endoscopic groups (Table 2). Bakker et al. [12] reported fewer cases of new-onset multiple organ failure and pancreatic fistula after EN compared with SN. Similarly, van Brunschot et al. [13] showed a reduction in cardiovascular and persistent organ failure with EN, though not all comparisons reached statistical significance. Bang et al. [15] demonstrated significantly fewer enteral–pancreatic cutaneous fistulas in the EN group. Garg et al. [14] noted lower rates of fever but more frequent need for additional endoscopic procedures compared to MISN. Onnekink et al. [8] confirmed that EN reduced the incidence of pancreatic fistula compared with MISN (8% vs. 34%, *p* = 0.002).

Mortality varied across trials, with no consistent difference between endoscopic and surgical step-up strategies. Across the randomized studies reporting this outcome, endoscopic approaches resulted in 28 deaths among 196 patients (14.3%), while surgical or minimally invasive surgical approaches resulted in 19 deaths among 186 patients (10.2%). Because of the heterogeneity in study populations, interventions, and outcome definitions, these numbers are presented descriptively without formal statistical pooling. LOS varied, but some studies (e.g., Angadi et al. [16]) reported shorter hospitalizations with EN. Long-term outcomes, including endocrine and exocrine insufficiency, were largely similar across groups, although EN tended to reduce pancreatic enzyme dependence in some trials. QoL outcomes generally improved after both approaches, with no consistent long-term differences; however, isolated studies suggested early benefits in physical functioning scores for EN (Table 3).

Taken together, the available randomized evidence suggests that endoscopic necrosectomy provides comparable survival outcomes to surgical or minimally invasive surgical necrosectomy, while reducing selected post-procedural complications (notably pancreatic fistula and some forms of organ failure) and, in some studies, hospital stay. Long-term pancreatic function and QoL appear similar between approaches, although early QoL may favor EN.

## 4. Discussion

This systematic review of six RCTs comparing endoscopic and surgical necrosectomy demonstrates that endoscopic management achieves similar mortality outcomes while significantly reducing peri-procedural morbidity, particularly pancreatic fistula and organ failure. Some studies also reported shorter hospital stays and faster recovery with endoscopy [8,13,15,16]. Long-term pancreatic function and quality of life were comparable between the two modalities.

Across the RCTs included in this review, mortality was comparable between the two strategies, with endoscopic approaches showing 28 deaths among 196 patients (14.3%) and surgical or minimally invasive surgical approaches showing 19 deaths among 186 patients (10.2%). These findings are in line with the meta-analysis by Haney et al. [17], which included three randomized trials (190 patients) and similarly found no significant difference in mortality between endoscopic and surgical step-up management.

Moreover, Mohamadnejad et al. [18], in a comprehensive meta-analysis including 11,807 patients, reported substantially lower mortality with endoscopic drainage (3%) compared with minimally invasive surgery (8%), surgical step-up approaches (13%), and especially open surgery (22%). In comparative analyses, endoscopic therapy was associated with a significantly reduced risk of death compared with both open (RR 0.30) and minimally invasive surgical approaches (RR 0.40) [18]. More recently, a 2024 network meta-analysis by Tan et al. [19] confirmed these trends, identifying upfront endoscopic necrosectomy as the strategy with the lowest mortality across all treatment modalities, followed by endoscopic step-up strategies, whereas open necrosectomy consistently ranked worst.

The management of infected necrotizing pancreatitis remains a significant clinical challenge, requiring a balance between adequate source control and minimization of iatrogenic morbidity. Historically, open necrosectomy was the mainstay of therapy but was associated with unacceptably high complication rates, including multiple organ failure, pancreatic fistulas, long-term exocrine and endocrine insufficiency, and impaired quality of life [4,20,21]. The evolution toward minimally invasive approaches has reshaped current paradigms, with both endoscopic and surgical step-up strategies demonstrating superior safety profiles compared to open surgery [4,22].

Recent evidence further supports these observations. The meta-analysis by Haney et al. [17] demonstrated that, while mortality did not differ significantly between approaches, endoscopic treatment was associated with substantially fewer major complications, including a reduced risk of new-onset multiple organ failure, visceral perforation or enterocutaneous fistula, and pancreatic fistula, as well as a shorter hospital stay. Similarly, the meta-analysis by Tang et al. [23] confirmed that endoscopic management significantly lowered the rates of major complications, organ failure, surgical site infection, fistula or perforation, and pancreatic fistula, again without differences in mortality. These findings are supported by the meta-analysis by Wu et al. [24], which also showed that endoscopic therapy resulted in fewer postoperative complications, markedly less new-onset organ failure, and lower rates of pancreatic fistula than minimally invasive surgical approaches. Collectively, these high-quality analyses highlight a consistent pattern: although survival remains comparable, endoscopic strategies provide a safer peri-procedural profile with significantly reduced morbidity across multiple clinically relevant endpoints.

Another important observation across the included trials is the variability in the timing of intervention. Although international guidelines generally recommend delaying drainage for at least four weeks after the onset of acute pancreatitis to allow wall maturation, not all studies adhered strictly to this timeframe. While Bakker et al. [12] and van Brunschot et al. [13] performed drainage around the fifth to sixth week, Onnekink et al. [8] reported earlier endoscopic interventions—often within two to six weeks—whereas Garg et al. [14] included patients drained at much later stages. This heterogeneity reflects the absence of a standardized definition for “early” versus “delayed” intervention and suggests that clinical factors such as infection, organ failure, or symptomatic burden frequently influence timing.

These findings align with the progressive shift from open and minimally invasive surgical necrosectomy toward endoscopic step-up approaches as first-line therapy. Endoscopic necrosectomy reduces tissue trauma and systemic inflammation, thereby improving short-term recovery while maintaining equivalent survival. However, treatment selection should remain individualized, as patients with extensive, multiloculated, or retroperitoneally extended necrosis may still require surgical or percutaneous drainage.

Over the last decade, randomized trials have increasingly positioned endoscopic necrosectomy as a preferred first-line strategy for walled-off pancreatic necrosis when technically feasible. While survival outcomes remain comparable to surgical approaches, endoscopic therapy consistently offers a safer peri-procedural profile, with fewer pancreatic or enteric fistulas and lower rates of new-onset organ failure [8,12,13,15]. These advantages translate into earlier recovery and, in some studies, better short-term quality-of-life measures [8,13].

Importantly, the benefits of endoscopic management appear to be predominantly early; long-term pancreatic function—both endocrine and exocrine—does not differ substantially from surgical techniques [5]. This underscores that treatment selection should be individualized, balancing the peri-procedural safety of endoscopy with the broader clinical and anatomical context. Patients with extensive, multiloculated, or laterally extending necrosis may still require percutaneous or surgical debridement, either alone or in combination [4,6].

Collectively, the evidence supports an “endoscopy-first” paradigm while reaffirming the ongoing role of surgery as a complementary option in complex or refractory cases.

The present results are consistent with prior meta-analyses and observational studies that support an endoscopy-first approach in suitable cases of walled-off necrosis. For instance, Zeng et al. [7] and Pavlek et al. [19] similarly reported reduced morbidity and comparable long-term pancreatic function with endoscopic drainage. These converging data reinforce the transition toward minimally invasive, multidisciplinary management of necrotizing pancreatitis.

From a practical standpoint, these findings support a multidisciplinary ‘endoscopy-first’ strategy with early escalation to surgery only when necessary. Decision-making should consider anatomical feasibility, extent of necrosis, and local expertise. The step-up philosophy—progressing from drainage to debridement—remains the cornerstone of safe, effective management.

Strengths and limitations of available evidence

This systematic review has several strengths. By including only randomized controlled trials and adhering to PRISMA 2020 guidelines, it provides a rigorous synthesis of high-level evidence comparing endoscopic and surgical approaches in necrotizing pancreatitis. The analysis considered both short- and long-term outcomes, including complications, mortality, pancreatic function, and quality of life, ensuring clinical relevance for decision-making.

Nonetheless, certain limitations should be acknowledged. Only six RCTs met the inclusion criteria, and some enrolled relatively small patient samples, which reduces the precision of individual effect estimates. In addition, heterogeneity existed in study design, patient selection, timing of interventions, and outcome definitions. These differences, inherent to the available randomized literature, limited the feasibility of a fully standardized quantitative meta-analysis.

Variability in the reporting of complications—such as major morbidity, organ failure, and fistula formation—also influenced the analytic approach. We extracted all outcomes exactly as reported, but the level of detail was not always sufficient to allow complete retrospective harmonization according to standardized classifications. As a result, a narrative synthesis offered a more reliable evaluation of the evidence than an artificial pooling of non-uniform endpoints.

Interpretation of procedural outcomes is further influenced by the step-up strategy adopted in most trials, where drainage constitutes the first intervention and necrosectomy is performed only when necessary. Because outcomes were not consistently stratified according to whether patients underwent drainage alone or progressed to necrosectomy, separate subgroup analyses were not feasible. This reflects the intrinsic characteristics of step-up trial designs rather than a limitation of the review itself.

Finally, effect modifiers such as necrosis etiology or anatomical complexity were not uniformly reported across studies and could not be analyzed systematically. Their potential influence on outcomes should therefore be considered when interpreting the findings. Overall, despite these limitations—which largely reflect the characteristics of the existing RCTs—the available evidence provides consistent and clinically valuable insights into the relative merits of endoscopic and surgical approaches. 

Future directions

There is a need for pragmatic trials and registries that capture the spectrum of real-world practice, including patients undergoing combined endoscopic–surgical strategies. Studies should also focus on long-term functional outcomes, particularly endocrine and exocrine pancreatic insufficiency, as well as cost-effectiveness and health-related quality of life. Furthermore, advances in endoscopic devices, lumen-apposing stents, and hybrid endoscopic–surgical platforms may expand the applicability of minimally invasive approaches and reduce the need for multiple interventions.

## 5. Conclusions

Evidence from RCTs strongly supports endoscopic necrosectomy as the preferred first-line therapy due to lower morbidity, particularly regarding pancreatic fistula and organ failure, with comparable mortality to surgical step-up approaches. However, surgical debridement remains indispensable for cases where endoscopic therapy fails or is anatomically inadequate. Ultimately, individualized multimodal management, guided by evolving evidence and patient-specific factors, represents the cornerstone of modern care for necrotizing pancreatitis.

## Figures and Tables

**Table 1:** Summary of Randomized Controlled Trials Comparing Endoscopic and Surgical Management of Necrotizing Pancreatitis.

Author	Year	Recruitment Period	Population Size *	Population	Intervention °	Comparison °	Outcome
Bakker [12]	2012	2008–2010	34	Infected necrotizing pancreatitis	EN = 10	SN = 10	Proinflammatory response and clinical outcome
van Brunschot S [13]	2018	2011–2015	418	Infected necrotizing pancreatitis	EN = 51	SN = 47	Major complications or death during 6-month follow-up
Bang JY [15]	2019	2014–2017	168	Infected necrotizing pancreatitis	MISN = 32	EN = 34	Major complications (new-onset multiple organ failure, new-onset systemic dysfunction, enteral or pancreatic-cutaneous fistula, bleeding and perforation of a visceral organ) or death during 6 months of follow-up
Garg PK [14]	2020	2010–2015	182	Symptomatic pseudocyst/WON (<30% necrotic debris of the cyst volume)	MISN = 30	EN = 30	Compare endoscopic and laparoscopic internal drainage of pseudocyst/walled-off necrosis following AP
Angadi S [16]	2021	2016–2018	145	WON >4 weeks duration with size >6 cm in diameter and having good interface with the stomach or duodenum, infected or symptomatic	MISN = 20	EN = 20	Compare laparoscopic drainage with endoscopic drainage for the resolution of WON without need of re-intervention
Onnekink AM [8]	2022	2011–2015	98	Infected necrotizing pancreatitis	EN = 51	MISN = 47	Mortality and major complications

* Population size refers to the overall cohort screened or enrolled in each study; ° the number of individuals actually allocated to endoscopic vs. surgical treatment.

**Table 2:** Comparison of Early Morbidity and Procedural Complications Between Endoscopic and Surgical Management of Necrotizing Pancreatitis.

Author	Post-Procedural Complications	
Bakker [12]	New-onset multiple organ failure	SN = 5 (50%) vs. EN = 0
	Intra-abdominal bleeding	SN = 0 vs. EN = 0
	Enterocutaneous fistula	SN = 2(20%) vs. EN = 0
	Pancreatic fistula	SN = 7(70%) vs. EN = 1 (10%)
van Brunschot S [13]	New-onset organ failure	
	Pulmonary	EN = 4 (8%) vs. SN = 7 (15%) RR = 0.53 (0.16–1.68); *p* = 0.27
	Persistent pulmonary	EN = 4 (8%) vs. SN = 5 (11%) RR = 0.74 (0.21–2.58); *p* = 0.63
	Cardiovascular	EN = 3 (6%) vs. SN = 9 (19%) RR = 0.31 (0.09–1.07); *p* = 0.045
	Persistent cardiovascular	EN = 2 (4%) vs. SN = 8 (17%) RR = 0.23 (0.05–1.03); *p* = 0.032
	Renal	EN = 2 (4%) vs. SN = 6 (13%) RR = 0.31 (0.07–1.45); *p* = 0.11
	Persistent renal	EN = 2 (4%) vs. SN = 6 (13%) RR = 0.31 (0.07–1.45); *p* = 0.11
	Single organ failure	EN = 7 (14%) vs. SN = 13 (28%) RR = 0.50 (0.22–1.14); *p* = 0.087
	Persistent single organ failure	EN = 6 (12%) vs. SN = 11 (23%) RR = 0.50 (0.20–1.25); *p* = 0.13
	Multiple organ failure	EN = 2 (4%) vs. SN = 6 (13%) RR = 0.31 (0.07–1.45); *p* = 0.11
	Persistent multiple organ failure	EN = 2 (4%) vs. SN = 5 (11%) RR = 0.37 (0.08–1.81); *p* = 0.20
	Bleeding (requiring intervention)	EN = 11 (22%) vs. SN = 10 (21%) RR = 1.01 (0.47–2.17); *p* = 0.97
	Perforation of a visceral organ or interocutaneous fistula (requiring intervention)	EN = 4 (8%) vs. SN = 8 (17%) RR = 0.46 (0.15–1.43); *p* = 0.17
	Pancreatic fistula	EN = 2/42 (5%) vs. SN = 13/41 (32%) RR = 0.15 (0.04–0.62); *p* = 0.0011
Bang JY [14]	New-onset multiple organ failure	EN = 2(5.9) vs. MISN = 3(9.4) RR = 0.63(0.11–3.51) *p* = 0.668
	New-onset multiple systemic dysfunction	EN = 0 vs. MISN = 1(3.1) *p* = 0.485
	Enteral-pancreatic cutaneous fistula	EN = 0 vs. MISN = 9(28.1) *p* = 0.001
	Visceral perforation	EN = 0 vs. MISN = 0 *p* = 0.999
	Intra-abdominal bleeding	EN = 0 vs. MISN = 3(9.4) *p* = 0.108
Garg PK [15]	Clavien Dindo class I	
	Delayed gastric emptying	MISN = 3 (10%) vs. EN = 1 (3.3%); *p* = 0.6
	Surgical site infection	MISN = 5 (16.6%)
	Enterocutaneous fistula	MISN = 1 (3.3%)
	Stent migration	EN = 1 (3.3%)
	Clavien Dindo class II	
	Blood transfusion	MISN = 8 (26.6%) vs. EN = 3 (10%); *p* = 0.19
	Fever	MISN = 9 (30.0%) vs. EN = 19 (63·3%); *p* = 0.01
	Pneumonia	MISN = 2 (6.6%) vs. EN = 0; *p* = 0.5
	Clavien Dindo class III	
	Gastric perforation with peritonitis	MISN = 0 vs. EN = 1 (3.3%); *p* = 0.9
	Need for additional procedures	
	Endoscopic drainage/lavage	MISN = 3 vs. EN = 15; *p* = 0.0001
	Percutaneous drainage	MISN = 1 vs. EN = 2
	Laparoscopic drainage	EN = 2
	Clavien Dindo class IVa	
	Respiratory failure	MISN = 1 (3.3%) vs. EN = 1 (3.3%); *p* = 1
	Septic shock	MISN = 1 (3.3%) vs. EN = 0; *p* = 0.9
	Peritonitis with shock	MISN = 0 vs. EN = 1 (3.3%); *p* = 0.9
Angadi S [16]	Clavein Dindo class I	
	Surgical site infection	MISN = 0
	Enterocutaneous fistula	MISN = 0
	Stent migration	EN = 0
	Clavien Dindo class II	*p* = 0.14
	Bleeding	MISN = 1 vs. EN = 1
	Secondary Infection	MISN = 5 vs. EN = 4
	Clavien Dindo class III	
	Perforation of hollow viscus	MISN = 1 vs. EN = 0
	Clavien Dindo Class IV	MISN = 0 vs. EN = 0
Onnekink AM [8]	Considering EN = 51 and MISN = 47	
	New-onset organ failure	EN = 11(22) vs. MISN = 15(32) RR = 0.68 (0.35–1.32); *p* = 0.263
	Multiple new-onset organ failure	EN = 4(8) vs. MISN = 6(13) RR = 0.61 (0.19–2.04); *p* = 0.513
	Bleeding requiring intervention	EN = 13(26) vs. MISN = 11(23) RR = 1.09 (0.54–2.19); *p* = 1
	Perforation or enterocutaneous fistula requiring intervention	EN = 6(12) vs. MISN = 11(23) RR = 0.5(0.20–1.25); *p* = 0.182
	Incisional hernia	EN = 4(8) vs. MISN = 4(9) RR = 0.92 (0.24–3.48); *p* = 1
	Biliary stricture	EN = 3(6) vs. MISN = 4(9) RR = 0.69 (0.16–2.93); *p* = 0.707
	Wound infection	EN = 3(6) vs. MISN = 4(9) RR = 0.69 (0.16–2.93); *p* = 0.707
	Pancreatic fistula	EN = 4(8) vs. MISN = 16(34) RR = 0.23 (0.08–0.64); *p* = 0.002

**Table 3:** Comparative Outcomes of Endoscopic Versus Surgical Interventions in Necrotizing Pancreatitis: Mortality, Morbidity, and Quality of Life.

Author	Mortality	LOS (Days)	Long-Term Complications	QoL After Intervention
Bakker [12]	SN = 4 (40%) EN = 1 (10%)	SN = 36 (17–74) EN = 45 (12–69)	New-onset diabetes SN = 3 (50%) vs. EN = 2 (22%) Use of pancreatic enzymes SN = 3 (50%) EN = 0 Persisting fluid collection SN = 3 (50%) vs. EN = 2 (22%)	-
van Brunschot S [13]	EN = 9 (18%) SN = 6 (13%)	EN = 35 (19–85) SN = 65 (40–90)	Exocrine insufficiency Use of enzymes EN = 16/42 (38%) vs. SN = 13/41 (32%) RR = 1.20 (0.66–2.17); *p* = 0.54 Fecal elastase <200 mg/g EN = 22/42 (52%) vs. SN = 19/41 (46%) RR = 1.13 (0.73–1.75); *p* = 0.58 Steatorrhea EN = 6/42 (14%) vs. SN = 7/41 (17%) RR = 0.84 (0.31–2.28); *p* = 0.73 Endocrine insufficiency EN = 10/42 (24%) vs. SN = 9/41 (22%) RR = 1.08 (0.49–2.39); *p* = 0.84	QALYs EN = 0.2788 (0.2458–0.3110) vs. SN = 0.2988 (0.2524–03398) Mean difference = −0.0199 (−0.0732–0.0395)
Bang JY [15]	EN = 3 (8.8) MISN = 2 (6.3) RR = 1.41 (0.25–7.91); *p* = 0.999	Median (IQR) EN = 14 (6–22) MISN = 18.5 (11.5–29.5)	New onset diabetes EN = 6 (27.3) vs. SD = 9 (36.0) RR = 0.76 (0.32–1.79); *p* = 0.522 New diagnosis of pancreatic insufficiency EN = 29 (85.3) vs. MISN = 28 (87.5) RR = 0.97 (0.80–1.18); *p* = 0.999	Quality of life at 3-month follow-up EN = MCS: 0.22 (9.18–8.87) *p* = 0.962 EN = PCS: 5.29 (0.27–10.3) *p* = 0.039
Garg PK [14]	EN = 0 vs. MISN = 0	MISN = 7 (4–52) EN = 8 (3–69) *p* = 0.1	-	-
Angadi S [16]	MISN = 0 vs. EN = 0	MISN = 6 (5–9) EN = 4 (4–8) *p* = 0.037	-	-
Onnekink AM [8]	15 of 51 patients (29%) in the endoscopy group and 7 of 47 patients (15%) in the surgery group died (RR, 1.89; 95% CI, 0.89–4.42). *p*-value 0.616	Median (IQR) EN = 52 (27–94) MISN = 72 (50–112) *p* = 0.090	Comparing EN = 36 vs. MISN = 40 Endocrine pancreatic insufficiency (HbA1c) EN = 16 (44) vs. MISN = 16 (40) RR = 1.11 (0.66–1.88); *p* = 0.817 Exocrine pancreatic insufficiency FE-1 < 200 mg/g EN = 12/27 (44) vs. MISN = 12/30 (40) RR = 1.11 (0.61–2.04); *p* = 0.792 Enzyme use at long-term follow-up EN = 11/36 (31) vs. MISN = 12/40 (30) RR = 1.02 (0.51–2.02); *p* = 0.792	Comparing EN = 29 vs. MISN = 30 SF-36 PCS EN = 45 ± 11 vs. MISN = 47 ± 10 *p* = 0.475 MCS EN = 48 ± 12 vs. MISN = 52 ± 10 *p* = 0.152 EQ-5D EN = 0.80 ± 0.23 vs. MISN = 0.86 ± 0.17 *p* = 0.237 Health state score EN = 72 ± 18 vs. MISN = 77 ± 13 *p* = 0.263

## Data Availability

Due to the nature of the study, there is no other data to share.

## Abbreviations

The following abbreviations are used in this manuscript:

AP	Acute pancreatitis
CI	Confidence interval
D1–D5	Domains of bias
EUS	Endoscopic ultrasound
EN	Endoscopic necrosectomy
EQ-5D	EuroQol-5 Dimension questionnaire
HbA1c	Hemoglobin A1c
IQR	Interquartile range
LOS	Length of stay
MISN	Minimally invasive surgical necrosectomy
MCS	Mental Component Summary (SF-36)
PCS	Physical Component Summary (SF-36)
PRISMA	Preferred Reporting Items for Systematic Reviews and Meta-Analyses
QoL	Quality of life
QALY/QALYs	Quality-adjusted life year(s)
RCT/RCTs	Randomized controlled trial(s)
RoB 2	Revised Cochrane Risk-of-Bias Tool for Randomized Trials
RR	Relative risk
SF-36	Short Form-36 Health Survey
SN	Surgical necrosectomy
VARD	Video-Assisted Retroperitoneal Debridement
WON	Walled-off necrosis
